# Transition from pediatric to adult neurology care in Duchenne muscular dystrophy: a national survey of patient and physician experiences

**DOI:** 10.1186/s13023-026-04454-8

**Published:** 2026-06-22

**Authors:** Gulcin Akinci, Eray Ontas, Hacer Durmus, Haluk Topaloglu

**Affiliations:** 1 Pediatric Neurology, Dr. Behcet Uz Children’s Hospital, Izmir Faculty of Medicine, University of Health Sciences Turkey, Izmir, Turkey; 2 Division of Epidemiology, Department of Public Health, Ankara University Faculty of Medicine, Ankara, Turkey; 3 Department of Neurology, Istanbul Faculty of Medicine, Istanbul University, Istanbul, Turkey; 4 Pediatric Neurology, Yeditepe University, Istanbul, Turkey

**Keywords:** Duchenne muscular dystrophy, Transition of care, Pediatric neurology, Adult neurology, Pediatric to adult care, Neuromuscular diseases, Patient and caregiver perspectives

## Abstract

**Background:**

Effective transition from pediatric to adult care is essential in Duchenne muscular dystrophy (DMD), but evidence on how transition is delivered in everyday practice remains limited. In Turkey, no clinical framework specifies how transition should occur, although national regulation requires completion of transfer by age 23. This study aimed to describe current practice, identify barriers and facilitators from physician and patient perspectives, and compare findings with international frameworks to inform a national guideline.

**Methods:**

As part of the AIM-DMD (National plan of action to raise Awareness and Improve Medical care of Duchenne Muscular Dystrophy) initiative, we conducted a national cross-sectional survey between June and December 2025, comprising parallel sub-studies of physicians and patients with DMD and their caregivers. Two structured online questionnaires were developed, guided by the Got Transition Six Core Elements framework and international Delphi consensus statements. The physician survey was distributed through professional networks; the patient and caregiver survey through the DMD Families Association and neuromuscular reference centers.

**Results:**

Sixty-two physicians (43 pediatric and 19 adult neurologists) from 28 cities and 48 patients and caregivers from 24 of these cities responded. Only 17.7% of physicians reported a systematic institutional transition program, 38.7% prepared individualized transition plans, and 9.7% described true readiness assessment. Joint pretransition consultations were held regularly by only 6.5% of physicians. Among transitioned patients, 72.4% first heard about transition at age 17 or later, and 62.1% considered their preparation inadequate. All physicians reported transferring medical data at transition, most commonly through institutional electronic health records (80.6%); however, 41.4% of patients and caregivers were unaware that any information had been conveyed to the adult team. Physicians, patients, and caregivers all identified the lack of a formal transition protocol, joint pediatric and adult consultations, multidisciplinary integration, and a designated coordinator role as priority areas for improvement.

**Conclusions:**

DMD transition care across Turkey lacks consistent structure. Key gaps include limited readiness assessment, delayed discussions, poor patient and caregiver awareness of the transition process, and lack of coordinated multidisciplinary structures. Our study identified common priorities raised by physicians, patients, and caregivers that need to be improved and could inform the development of standardized protocols.

**Supplementary information:**

The online version contains supplementary material available at https://doi.org/10.1186/s13023-026-04454-8.

## Background

Advances in disease-modifying treatments and medical care have allowed more adolescents and young adults with chronic childhood-onset conditions to live into adulthood, making the transition from pediatric to adult care a central part of their long-term management [1, 2]. Transition is more than a single referral. It requires early preparation, sustained collaboration between pediatric and adult teams, structured information transfer, and post-transfer follow-up [3]. Poorly structured transitions are associated with medical complications, reduced adherence, interrupted follow-up, more emergency visits, and greater psychosocial distress; well-planned transitions are associated with better adherence, higher satisfaction, and more stable health in adulthood [4–6]. Rare diseases bring these problems into sharper focus, since they typically begin in childhood and depend most heavily on a coordinated transition at the interface between pediatric and adult care [7].

Duchenne muscular dystrophy (DMD) is the most common severe muscular dystrophy of childhood, an X-linked disorder affecting roughly 1 in 3,500 to 5,000 male births [8, 9]. Progressive skeletal muscle degeneration leads to loss of ambulation by early adolescence, followed by respiratory and cardiac complications that typically require assisted ventilation during the second or third decade [8]. Corticosteroids, gene-based treatments, and cardiorespiratory supportive care have extended life expectancy into the third and fourth decades [9, 10], and a growing number of individuals with DMD now need coordinated multidisciplinary follow-up throughout adulthood [11]. Progressive disability, lifelong disease-modifying therapy, and a shortage of adult neuromuscular specialists make transition particularly difficult in this population [12].

International frameworks address some of these challenges. The Six Core Elements of Health Care Transition, developed by Got Transition and endorsed in 2018 by the American Academy of Pediatrics, the American Academy of Family Physicians, and the American College of Physicians, recommends introducing transition planning between ages 12 and 14 and completing transfer by age 26, with written plans beginning at age 14 [3]. A 2025 international Delphi consensus on DMD recommends that young people and their families be made aware of transfer plans before age 15, with transition completed by age 25 [13]. Actual practice diverges from these recommendations: in Canada, Germany, and Japan, transfer typically occurs around age 18 [13–15]; only 17% of US youth with special health care needs receive all recommended transition components [16]; and only 23% of young adults with DMD or BMD in a US survey had a written transition plan [17]. How DMD transition is carried out in everyday practice across countries remains poorly characterized [18].

In Turkey, Ministry of Health Circular No. 2018/30 establishes the broad framework: ages 18 to 23 are designated as a transition period for patients with childhood-onset chronic diseases, with handover to be completed by age 23. The circular permits pediatric subspecialists to continue follow-up beyond that age when no adult specialist is available, acknowledging the uneven distribution of adult subspecialty coverage across the country. Disease-specific guidance, structured readiness assessment, and post-transfer follow-up are not addressed, and no neuromuscular-specific national transition protocol exists. How the circular is applied in clinical practice, particularly for rare conditions such as DMD, has not been described.

This study was conducted within the AIM-DMD initiative (National plan of action to raise Awareness and Improve Medical care of Duchenne Muscular Dystrophy), a 36-month national program bringing together clinicians, rehabilitation professionals, patient associations, and families involved in DMD care in Turkey. The aim of the present survey was to describe current transition practice, identify barriers and facilitators from physician and patient perspectives, and compare findings with international frameworks. Establishing this baseline and identifying the priorities shared by physicians, patients, and families is a necessary first step toward the targeted interventions planned within AIM-DMD and toward developing a national framework that can guide DMD transition care more consistently across Turkey.

## Methods

### Study design and setting

Conducted within the AIM-DMD (National plan of action to raise Awareness and Improve Medical care of Duchenne Muscular Dystrophy) initiative, this was a cross-sectional, multicenter, descriptive study with a mixed-methods design, comprising two parallel sub-studies: a physician survey targeting pediatric and adult neurologists involved in the care of individuals with DMD, and a patient and caregiver survey targeting individuals with DMD and their primary caregivers. The study was conducted across Turkey between June 2025 and December 2025. Ethical approval was granted by the Ankara University Faculty of Medicine Human Research Ethics Committee on 24 June 2025 (approval number 2025/481). The study was designed and reported in accordance with the Strengthening the Reporting of Observational Studies in Epidemiology (STROBE) guidelines for cross-sectional studies.

### Participants and eligibility

For the physician sub-study, eligible participants were pediatric or adult neurology specialists actively involved in the management of individuals with DMD at neuromuscular reference centers in Turkey.

For the patient and caregiver sub-study, eligible participants were individuals aged 12 years or older with a diagnosis of DMD and their parents or caregivers. Participants with a diagnosis of Becker muscular dystrophy and patients under 12 years of age were excluded. The survey could be completed by the patient or by a parent or caregiver on the patient’s behalf; respondents identified their role at the start of the survey. For participants aged 18 years or older completing the survey themselves, informed consent was obtained directly from the participant. For patients under 18 years, informed consent was provided by the parent or caregiver.

### Survey development

Two structured questionnaires were developed for this study, one for physicians and one for patients and caregivers. Survey content was informed by the Got Transition Six Core Elements of Health Care Transition framework [3] and by existing literature on transition in neuromuscular disease, including recent Delphi consensus statements [13, 19]. Draft items were reviewed by pediatric and adult neurologists with expertise in neuromuscular disease and by representatives of the DMD Families Association to assess clarity, relevance, and face validity; items were revised based on this feedback before distribution. Both questionnaires were administered in Turkish and are available as Supplementary Material, with English translations provided.

The physician survey comprised 32 items in eight sections: demographics and institutional characteristics; transition policy awareness (including awareness of Ministry of Health Circular 2018/30) and institutional transition practices; monitoring and follow-up; readiness assessment; transition planning; transfer to adult care and transition completion; clinical barriers to transition; and recommendations for improvement. Barriers were rated on a 5-point Likert scale (strongly disagree to strongly agree) across 15 items, and recommendations on a 5-point importance scale (not important to very important) across 6 items. A separate item assessed overall satisfaction with the institutional transition process on a three-level ordinal scale (completely, partially, not satisfied). Estimated completion time was 15 to 20 minutes.

The patient and caregiver survey comprised 50 items in 17 sections covering demographics and anonymous identifiers; educational and occupational status; family history; primary care involvement; medication knowledge; patient-only consultations with the pediatric neurologist; current clinical follow-up; transition-related communication and exposure to the adult service; perceived adequacy of transition preparation; the transition process itself; psychological support; transition-related concerns; self-care and medication responsibility; information sources; future goals; suggestions for improving transition; and comparative evaluation of pediatric versus adult follow-up. Response formats were single-select, multi-select, and open-ended. Pre- and post-transition respondents completed the same instrument, with branching logic directing each to the relevant sections. Estimated completion time was 15 to 20 minutes. Patient and caregiver responses regarding information transfer reflected respondent experience and awareness of the handover, rather than independent verification of documentation transfer.

### Data collection

Both questionnaires were administered as structured online forms (Google Forms) in Turkish.

The physician survey was distributed through the institutional networks of the Neuromuscular Research Society, Turkey and the Turkish Neurological Society. Society representatives shared the survey with member physicians involved in DMD care, who were asked to forward it to colleagues within their centers. Because recruitment relied on this cascade through professional networks, the number of physicians who received the survey link could not be tracked, and a formal response rate is not reported.

The patient and caregiver survey was distributed through the DMD Families Association and through neuromuscular reference centers, where physicians shared the survey link with their patients. The eligibility criterion of patient age ≥ 12 years was stated in the survey invitation; however, some ineligible responses (from families of younger patients) were nonetheless received and excluded during screening. Because the invited population could not be restricted to eligible recipients, an exact response rate cannot be calculated for this sub-study. To detect duplicate entries while preserving confidentiality, the survey captured the first two letters of the patient’s first name and surname, year of birth, and city of residence; duplicates were identified using these combined identifiers, and the most recent entry was retained.

Responses to both surveys were anonymous. Physicians provided their city of practice and the name of their center; patient and caregiver respondents provided their city of residence. Individual respondent names were not collected. Participation was voluntary, and incomplete surveys or responses without valid informed consent were excluded from analysis.

### Variables and outcome measures

The primary measures were the presence and characteristics of structured transition programs at participating centers, physician- and patient-reported barriers and facilitators to transition, and patient-reported experiences of the transition process. Physician-reported barriers (15 Likert-scale items) were collapsed into three categories for analysis: agreement (strongly agree, agree), neutral, and disagreement (disagree, strongly disagree). The six recommendation items were analyzed as continuous variables on a 1 to 5 scale. Physician satisfaction with the institutional transition process was analyzed on the three-level ordinal scale described above. Patient-reported measures covered the transition process, information transfer between pediatric and adult teams, care coordination, patient autonomy, and comparative perceptions of pediatric versus adult follow-up.

### Statistical analysis

Descriptive statistics were used to summarize the survey data. Categorical variables are presented as frequencies and percentages. Continuous variables are reported as medians with ranges (minimum to maximum), reflecting the expected non-normal distributions and limited sample size. Rating items are presented as mean scores ranked in descending order. No inferential statistical analyses were performed.

Given the exploratory and descriptive aim of the study, open-ended responses from both the physician and patient/caregiver surveys were analyzed using qualitative content analysis. Responses were reviewed systematically, coded according to recurring themes, and grouped into broader categories related to transition practices, perceived barriers, unmet needs, and suggestions for improvement. The frequency and percentage of responses within each thematic category were reported.

### Patient and public involvement

The DMD Families Association contributed to the design and distribution of the patient and caregiver survey and supported recruitment. Informal consultations with patients and families during the development phase informed the content and wording of survey items.

## Results

### Physician sub-study

#### Participant characteristics

Sixty-two physicians completed the survey; their characteristics are summarized in Table 1. The median age was 44 years (range 28 to 65), and 39 (62.9%) were women. Forty-three (69.4%) were pediatric neurologists and 19 (30.6%) were adult neurologists. Respondents practiced across 28 of Turkey’s 81 cities, including both major metropolitan areas and smaller centers, suggesting reasonable national coverage. Most worked in centers that included both pediatric and adult services (*n* = 51, 82.3%); 8 (12.9%) worked in pediatric-only hospitals and 3 (4.8%) in adult-only hospitals. Twenty-one physicians (33.9%) practiced at centers officially designated as Neuromuscular Disease (NMD) Centers by the Ministry of Health, 18 (29.0%) at Ministry-designated NMD Outpatient Clinics, and 22 (35.5%) at centers without a dedicated NMD structure. One respondent (1.6%) worked at a formally designated NMD center that was not actively functioning. Time per DMD outpatient visit (*n* = 61; one unrelated response excluded) was 15 to 30 minutes for 33 physicians (54.1%) and less than 15 minutes for 22 (36.1%); only 6 (9.8%) reported visits longer than 30 minutes.

**Table 1:** Physician respondents: demographic, professional, and institutional characteristics (*n* = 62)

Variable	n	%
***Age*** (years), median (range)	44 (28–65)	—
***Sex***		
Female	39	62.9
Male	23	37.1
***Specialty***		
Pediatric neurology	43	69.4
Adult neurology	19	30.6
***Center designation for neuromuscular disease care***		
Ministry of Health-designated Neuromuscular Disease Center	21	33.9
Neuromuscular Disease Outpatient Clinic (formally designated)	18	29.0
Routine pediatric neurology or neurology practice (no dedicated Neuromuscular Disease Center structure)	22	35.5
Designated Neuromuscular Disease Center, but not actively functioning	1	1.6
***Time allocated per DMD outpatient visit ***^***a***^		
Less than 15 minutes	22	36.1
15 to 30 minutes	33	54.1
More than 30 minutes	6	9.8

^a^ *n* = 61; one response was excluded as unrelated to a specific time interval

#### Transition policy awareness and institutional practices

Physician-reported institutional context and transition practices are summarized in Tables 2 and 3. Forty-four physicians (71.0%) were aware of Ministry of Health Circular 2018/30, which sets age 23 as the latest age for transfer of patients with childhood-onset chronic diseases to adult specialist services; 18 (29.0%) were unaware of this regulation. Only 11 physicians (17.7%) reported that their institution had a systematic transition program or guideline for childhood-onset neurological diseases; 41 (66.1%) reported no such program and 10 (16.1%) were unsure. When asked specifically how DMD transition is managed at their institution, 21 (33.9%) reported no specific procedure and 18 (29.0%) described reliance on personal communication between pediatric and adult teams. Smaller proportions reported providing contact information to patients and families (*n* = 8, 12.9%), periodic joint meetings between teams (*n* = 3, 4.8%), or periodic joint clinics (*n* = 2, 3.2%). Six physicians (9.7%) provided free-text responses describing locally developed alternatives such as transfer forms, in-house transition files, or discharge-summary-based handover (*n* = 4); simple referral to adult neurology at age 18 without a structured process (*n* = 1); and one adult neurologist who reported having received no DMD transfers to date. Four physicians (6.5%) were unaware of their institution’s approach.

**Table 2:** Physician-reported transition framework, timing, and patient engagement (*n* = 62)

Variable	n	%
***Policy awareness and institutional framework***		
Aware of the national Ministry of Health regulation on transition to adult care^a^	44	71.0
Institution has a systematic transition program for childhood-onset neurological diseases		
Yes	11	17.7
No	41	66.1
Don’t know	10	16.1
How DMD transition is currently managed at the institution		
No specific procedure	21	33.9
Personal communication between pediatric and adult teams	18	29.0
Contact information for adult services provided to patients/families	8	12.9
Periodic joint meetings between teams	3	4.8
Joint clinics	2	3.2
Other^b^	6	9.7
Don’t know	4	6.5
***Transition timing***		
Age at which transition is typically initiated at the institution		
Before 18 years	8	12.9
At 18 years	38	61.3
After 18 years	16	25.8
Ideal age for initiating transition, in physician’s view (*n* = 61)		
Before 18 years	23	37.7
At 18 years	26	42.6
After 18 years	12	19.7
Age at full transfer to adult neurology		
Before 18 years	1	1.6
At 18 years	33	53.2
After 18 years	22	35.5
Reported as an age range rather than a single age^c^	6	9.7
***Patient engagement and planning***		
Institution maintains a systematic registry or list of patients in the transition process		
Yes	17	27.4
No	38	61.3
Don’t know	7	11.3
Uses a tool, form, or interview to assess transition readiness^d^		
Yes	16	25.8
No	43	69.4
Did not respond	3	4.8
Conducts one-on-one transition discussions with the adolescent/young adult		
Yes	32	51.6
No	28	45.2
Not applicable or don’t know	2	3.2
Prepares an individualized transition plan	24	38.7
Includes patient and family perspectives in transition planning	48	77.4

^a^ Ministry of Health Circular 2018/30 sets age 23 as the latest age for transfer to adult specialist care for childhood-onset chronic diseases

^b^ Free-text descriptions included locally developed approaches such as transfer forms, in-house transition files, discharge summary-based handover, and simple referral to adult neurology at age 18; one adult neurologist reported having received no DMD transfers to date

^c^ Age ranges reported were 18–19 (*n* = 1), 18–20 (*n* = 2), 18–22 (*n* = 2), 18–23 (*n* = 1), and 19–20 (*n* = 1) years

^d^ Most respondents using “some tool” referred to transfer documentation rather than readiness assessment; only 6 (9.7%) described practices specifically aimed at assessing readiness

**Table 3:** Physician-reported coordination and post-transition follow-up (*n* = 62)

Variable	n	%
***Information transfer to adult services***		
Method of medical information transfer^a^		
Institutional electronic health record system	50	80.6
Physical document or discharge summary	38	61.3
Telephone	13	21.0
Other	4	6.5
No information transfer	0	0
***Collaboration between pediatric and adult teams***		
Joint adult–pediatric consultations before transition		
Regularly	4	6.5
Occasionally	32	51.6
Never	26	41.9
***Post-transition follow-up and outcomes (pediatric neurologists, n = 43)***		
Continues temporary follow-up after transition	24	55.8
Receives feedback from adult team	15	34.9
***Perceived rate of loss to follow-up after transition (n = 62)***		
Low	21	33.9
High	13	21.0
Unable to estimate	28	45.2
***Overall satisfaction with the current transition process***		
Completely satisfied	2	3.2
Partially satisfied	46	74.2
Not satisfied at all	14	22.6

^a^ Multi-select item; percentages may exceed 100%

#### Tracking, readiness assessment, and patient engagement

Systematic tracking of patients in transition was uncommon. Seventeen physicians (27.4%) reported that their institution maintained a list or software-based registry for this purpose; 38 (61.3%) reported no such tracking system and 7 (11.3%) were unsure. Readiness assessment activities were rare. Sixteen physicians (25.8%) reported using “some tool,” but free-text descriptions revealed that most referred to transfer documentation practices, including detailed epicrises, structured discharge summaries, and transfer forms (*n* = 10), rather than readiness assessment. Only 6 respondents (9.7%) described practices specifically aimed at assessing transition readiness, all involving informal interviews or inter-colleague discussions. No respondent referenced a standardized or validated readiness assessment instrument. Forty-three physicians (69.4%) reported using no tool, and three did not answer. Direct patient engagement during transition preparation was variable. About half of physicians (*n* = 32, 51.6%) reported meeting alone with the pediatric or adolescent patient during the preparation period, while 28 (45.2%) did not. One physician had not yet had a transition patient, and one was unsure.

#### Transition timing

Transition initiation was most commonly reported at age 18 (*n* = 38, 61.3%), with 8 physicians (12.9%) initiating earlier and 16 (25.8%) later. Full transfer to adult neurology was also most commonly completed at age 18 (*n* = 33, 53.2%); 22 physicians (35.5%) reported transfer at older ages, up to 25 years. Six physicians (9.7%) reported transfer as an age range rather than a single age, most commonly 18 to 20 years (*n* = 2) and 18 to 22 years (*n* = 2), with single responses for ranges of 18 to 19, 18 to 23, and 19 to 20 years.

#### Transition planning, information transfer, and post-transition follow-up

While 48 physicians (77.4%) reported including patient and family perspectives in transition planning, only 24 (38.7%) prepared a formal individualized transition plan. Joint pre-transition consultations between pediatric and adult neurology teams were held regularly by 4 physicians (6.5%), occasionally by 32 (51.6%), and never by 26 (41.9%). Information transfer at the time of transition was universal; all respondents reported transferring some form of information, most commonly through institutional electronic health records (*n* = 50, 80.6%) and written discharge summaries (*n* = 38, 61.3%); 13 respondents (21.0%) also used telephone communication. Among the 43 pediatric neurologists who answered post-transition follow-up items, 24 (55.8%) continued to follow their patients temporarily after transfer, but only 15 (34.9%) reported receiving feedback from the adult team. When asked about loss to follow-up after transition—assessed across all 62 physicians—21 physicians (33.9%) perceived the rate as low and 13 (21.0%) as high; 28 (45.2%) were unable to estimate it.

#### Perceived barriers to transition

Physician-perceived barriers are summarized in Table 4. The most strongly endorsed barriers were absence of a locally established transition program (74.2%), parental or caregiver preference to remain in the pediatric clinic (69.4%), lack of clinical environments in the adult system adapted for young adults with special needs (67.7%), insufficient awareness of the need for transition programs (67.7%), and the complexity of pediatric multisystem diseases (64.5%). Lack of financial support and insufficient training in transition were each endorsed by 61.3% of physicians. Communication-related barriers received moderate endorsement, including lack of communication between adult and pediatric teams (58.1%) and absence of multidisciplinary team structure in the adult system (53.2%). The least-endorsed items were the limited number of adult neurologists (25.8%) and difficulty accessing pediatric medical records (24.2%).

**Table 4:** Physician-perceived barriers to transition, ranked by proportion of agreement (*n* = 62) ^a^

Barrier	Agree, n (%)	Neutral, n (%)	Disagree, n (%)
Absence of a locally established transition program	46 (74.2)	9 (14.5)	7 (11.3)
Parental or caregiver preference to remain in the pediatric clinic	43 (69.4)	9 (14.5)	10 (16.1)
Lack of clinical environments in the adult system adapted for young adults with special needs	42 (67.7)	7 (11.3)	13 (21.0)
Insufficient awareness of the need for, and implementation of, transition programs	42 (67.7)	10 (16.1)	10 (16.1)
Complexity of pediatric multisystem diseases	40 (64.5)	11 (17.7)	11 (17.7)
Lack of financial support for transition programs	38 (61.3)	11 (17.7)	13 (21.0)
Insufficient training or education on transition	38 (61.3)	11 (17.7)	13 (21.0)
Lack of communication between adult and pediatric teams	36 (58.1)	9 (14.5)	17 (27.4)
Absence of a multidisciplinary team structure in the adult system	33 (53.2)	11 (17.7)	18 (29.0)
Inadequate guidelines for adult-period management of pediatric-onset diseases	32 (51.6)	13 (21.0)	17 (27.4)
Limited willingness of adult neurologists to accept these patients	29 (46.8)	13 (21.0)	20 (32.3)
Difficulty obtaining timely appointments in adult neurology	27 (43.5)	15 (24.2)	20 (32.3)
Insufficient knowledge of pediatric-onset diseases among adult neurologists	23 (37.1)	13 (21.0)	26 (41.9)
Limited number of adult neurologists	16 (25.8)	7 (11.3)	39 (62.9)
Difficulty accessing pediatric medical records	15 (24.2)	11 (17.7)	36 (58.1)

^a^ Likert responses were collapsed for presentation: “Agree” combines strongly agree and agree; “Disagree” combines strongly disagree and disagree. Items are ranked by proportion of agreement

Free-text responses drew a more specific distinction. While general adult neurologists were not perceived as scarce, the scarcity of adult neurologists with specific neuromuscular expertise was identified as a major concern, particularly outside major urban centers. Other concerns included difficulty obtaining appointments through adult neurology scheduling systems, the absence of dedicated transition clinics or structured pathways, the heavy workload that limits time available for complex patients in adult neurology, and medico-legal exposure for pediatric neurologists who continue to manage patients with adult-age complications when no formal handover pathway exists.

#### Recommendations for improving transition

Physicians rated six proposed recommendations on a five-point importance scale (1 = not important to 5 = very important). All six were strongly endorsed. Having a formal transition protocol received the highest rating (mean 4.44; 87.1% rated important or very important), followed by joint consultations between adult and pediatric neurology teams (4.39; 85.5%) and use of national transition recommendations (4.35; 83.9%). Inclusion of patient and caregiver views in transition planning (4.23; 79.0%), active participation of patients and families (4.16; 77.4%), and a dedicated transition coordinator (4.06; 75.8%) were also rated highly. Fewer than 4% of physicians rated any single recommendation as “not important.”

Free-text suggestions reinforced the rank-ordered recommendations and added operational detail. The most frequent suggestions were stronger communication and coordination between pediatric and adult neurology teams, including joint clinics and overlapping follow-up during the transition period. Specific operational models proposed included shared clinics beginning at ages 16 to 18 and requiring at least two joint consultations before handover. Other recommendations included dedicated time for transition within clinic schedules, transition coordinators or nurses, standardized handover documentation, and designated adult neuromuscular reference centers in each region. Several respondents emphasized that implementation would require structural support, including protected time for transition-related activities and administrative backing from hospital leadership.

Only 2 physicians (3.2%) reported full satisfaction with the current transition process at their institution; 46 (74.2%) were partially satisfied, and 14 (22.6%) reported no satisfaction at all.

### Patient and caregiver sub-study

#### Participant characteristics and flow

**Fig. 1:**
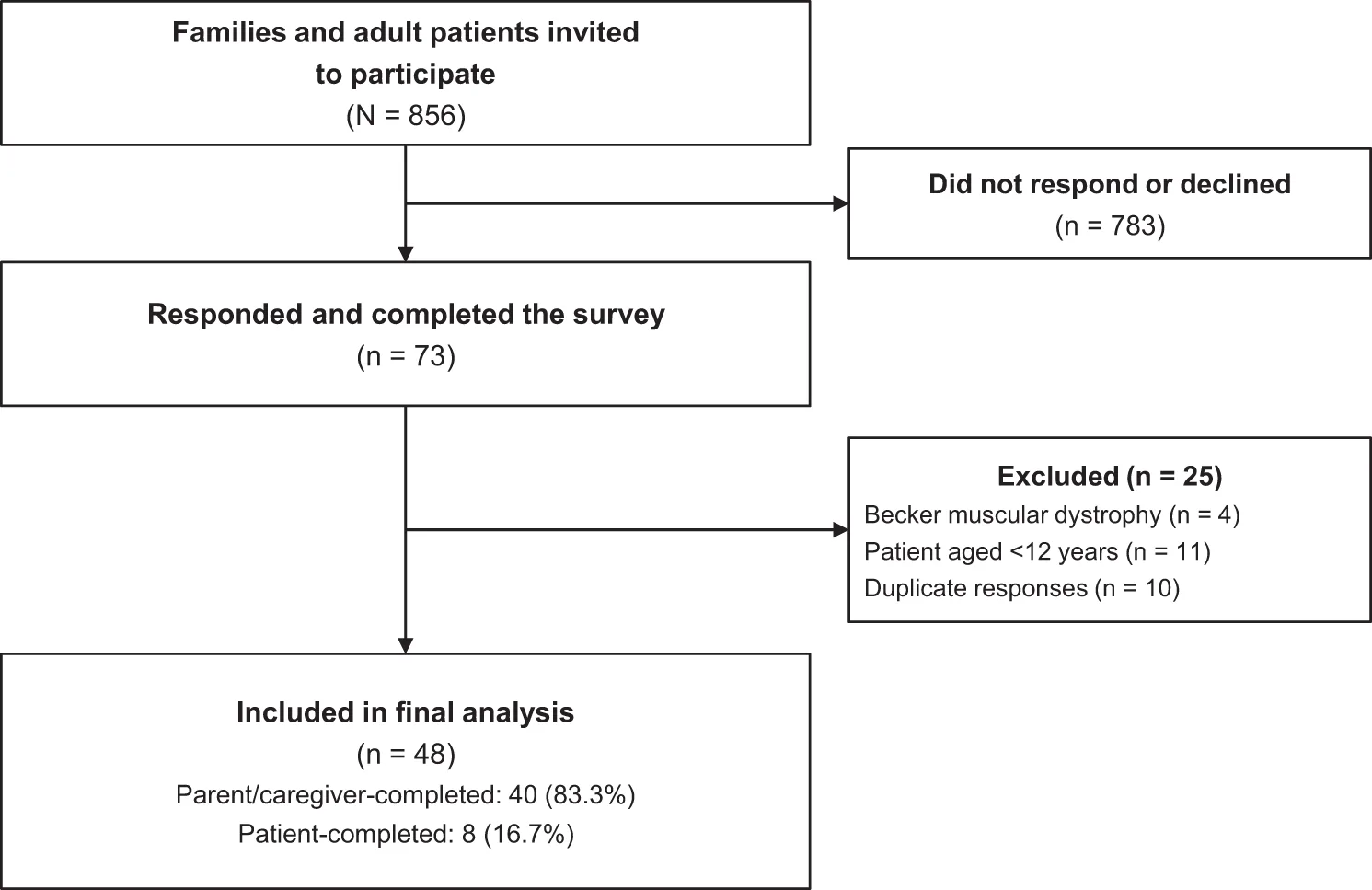
Patient and caregiver sub-study participant flow. Of 856 families and adult patients invited through the DMD Families Association and neuromuscular reference centers in Turkey, 73 responded (response rate 8.5%). Twenty-five were excluded: 4 with Becker muscular dystrophy, 11 with patient age under 12 years, and 10 duplicate entries (the most recent entry was retained for each). Forty-eight responses were analyzed: 40 (83.3%) completed by a parent or caregiver and 8 (16.7%) by the patient. The physician sub-study used cascade recruitment through professional society networks without exclusion criteria; all 62 responses received were included in the analysis

Of 856 families and adult patients invited to participate through the DMD Families Association and neuromuscular reference centers, 73 responded. Twenty-five responses were excluded: 4 for a diagnosis of Becker muscular dystrophy rather than DMD, 11 because the patient was under 12 years of age, and 10 for duplicate responses for the same patient (the most recent entry was retained). Forty-eight unique responses remained for analysis (Fig. 1). Surveys were completed by a parent or caregiver in 40 cases (83.3%) and by the patient directly in 8 cases (16.7%). Two respondents entered the caregiver’s birth date rather than the patient’s, leaving 46 valid age entries; the median patient age was 20 years (range 12 to 29). Responses came from 24 of Turkey’s 81 cities, most commonly Antalya (*n* = 10, 20.8%), Istanbul (*n* = 7, 14.6%), Ankara (*n* = 3, 6.3%), and Izmir (*n* = 2, 4.2%). Patient demographic and clinical characteristics are summarized in Table 5. A family history of DMD was reported in 25.0% of cases.

**Table 5:** Patient and caregiver respondents: demographic, health care access, and self-management characteristics (*n* = 48)

Variable	n	%
***Demographic and clinical characteristics***		
Patient age, years (*n* = 46), median (range)	20 (12–29)	
Family history of DMD	12	25.0
Respondent		
Parent or caregiver	40	83.3
Patient	8	16.7
***Health care access***		
Regular follow-up by a family physician	37	77.1
Current neurology follow-up setting		
Pediatric neurology	19	39.6
Adult neurology	29	60.4
***Patient autonomy and self-management***		
Patient knows medication names and doses	37	77.1
Ever had a one-on-one consultation with child neurologist (no family present)	13	27.1
Age at first one-on-one consultation (*n* = 12), years, median (range)	9.5 (5–18)	

#### Health care access and patient autonomy

Most patients were followed regularly by a family physician (*n* = 37, 77.1%); among these, the most commonly reported features were that the family physician prescribed medications (*n* = 23, 62.2%) and was knowledgeable about the patient’s disease (*n* = 17, 45.9%); fewer reported regular communication with the neurologist (*n* = 11, 29.7%) or advice on healthy lifestyle (*n* = 9, 24.3%).

At the time of the survey, 29 patients (60.4%) were followed in adult neurology and 19 (39.6%) in pediatric neurology; all 19 patients still in pediatric care were aged 12 to 17 years. Patient knowledge of medication names and doses was reported in 37 cases (77.1%), but only 13 patients (27.1%) had ever had a consultation with their child neurologist without a family member present; among those who reported an age (*n* = 12), the median age at first such consultation was 9.5 years (range 5 to 18).

#### Transition discussion among pediatric patients

Among the 19 patients still in pediatric care, only 3 (15.8%) reported that their child neurologist had discussed the transition to adult services; 11 (57.9%) reported no such discussion, and 5 (26.3%) were unsure. The three with whom transition had been discussed were aged 13, 13, and 16 years at the time of the discussion.

#### Transition experience

The transition experience of the 29 patients in adult neurology follow-up is summarized in Table 6. Pre-transition preparation was generally limited: only one patient (3.4%) had met with the adult neurology team before transfer, and transition was first discussed at age 17 or later in 21 cases (72.4%). Pre-transition information about the destination hospital, future care teams, or genetic counseling was provided to 37.9% to 44.8% of patients depending on the topic. Overall, 18 respondents (62.1%) considered their transition preparation inadequate.

**Table 6:** Patient-reported pre-transition preparation and transition process (*n* = 29 transitioned patients)

Variable	n	%
***Pre-transition preparation***		
Pre-transition meeting with adult neurology team	1	3.4
Age at which transition was first discussed		
12–14 years	3	10.3
15–16 years	1	3.4
≥17 years	21	72.4
Unknown	4	13.8
Topics discussed by the pediatric team with the patient before transfer^a^		
Which hospital the transition would be to	13	44.8
Which teams would be involved in future care	11	37.9
Genetic counseling	12	41.4
Perceived transition preparation as adequate	11	37.9
***Transition process and coordination***		
How transition was carried out		
No medical information was transferred to adult neurologist	12	41.4
Discussion with child neurologist	8	27.6
Structured/planned transition process	4	13.8
Informative letter from child neurologist	1	3.4
Family self-referred to adult services	1	3.4
Other^b^	3	10.3
Who coordinated the transition process		
Family (self-coordinated)	16	55.2
Child neurologist	10	34.5
Nurse	3	10.3
Psychological support during transition	5	17.2

^a^ Multi-select item; respondents could choose more than one topic. The original survey item asked whether the pediatric team had discussed each of these topics with the patient before transfer to the adult service

^b^ Other transition pathways (*n* = 3): one transition occurred within the same institution; in one case the pediatric file was directly handed to the adult neurologist within a neuromuscular disease center; and in one case transition was triggered by the expiration of the patient’s medical report period

Forty-one percent of patients and caregivers (*n* = 12) were unaware that any medical information had been conveyed to the adult neurologist, 27.6% (*n* = 8) recalled a conversation with the child neurologist, and 13.8% (*n* = 4) described a structured planned transition. Less common reports included an informative letter from the child neurologist (*n* = 1), family self-referral (*n* = 1), and three accounts that did not fit standard categories: one transition that occurred within the same institution, one in which the patient file was handed directly to the adult neurologist within a neuromuscular disease center, and one in which transition was forced by the expiration of the medical report period. Transition was managed primarily by families (*n* = 16, 55.2%), with the child neurologist coordinating in 34.5% of cases and a nurse in 10.3%. Psychological support during transition was received by 5 respondents (17.2%).

#### Post-transition autonomy, education, and information sources

Autonomy was variable: 41.4% of patients managed their own medications, 27.6% booked their own appointments, and 10.3% consulted the neurologist alone. Educational and vocational situations among the 29 transitioned patients varied widely (Supplementary Table 1). The most common situations were not working or not in education (37.9%), attending a special education or rehabilitation center (27.6%), and being enrolled in university (13.8% in standard programs and 6.9% with special education support). High school was the highest qualification completed by 48.3% of patients, with smaller proportions completing middle school (24.1%) or primary school (13.8%); only 4 patients (13.8%) had attained any post-secondary qualification. The most frequently used sources of disease and treatment information were patient groups and family associations (75.9%), internet resources (58.6%), the child neurologist (55.2%), and the adult neurologist (31.0%).

#### Comparison of pediatric and adult care periods

When comparing the two follow-up periods, 18 patients (62.1%) rated pediatric follow-up more favorably than adult follow-up, 10 (34.5%) considered them similar, and one (3.4%) preferred adult follow-up. Among 17 free-text explanations, the most commonly cited reasons for preferring pediatric care were physician communication (*n* = 6), information sharing (*n* = 4), coordination between teams (*n* = 3), appointment access (*n* = 2), and the limited availability of adult neurologists with NMD expertise (*n* = 2).

When asked about gaps in adult care that were not present in pediatric care, 11 of 25 respondents (44.0%) reported no specific gaps, often because they remained at a specialized neuromuscular center. Among those who identified gaps, the most common concern was loss of multidisciplinary care, particularly the absence of integrated cardiology and respiratory follow-up. A separate theme concerned communication style: pediatric clinicians were described as sensitive and explanatory, while adult clinicians were characterized as more direct but less attentive to emotional impact. The reported impact included emotional distress, difficulty accepting the change in care, and in some cases reduced engagement with health care or independent information seeking. Among 27 respondents who described the greatest difficulties encountered during transition, 9 (33.3%) reported no specific difficulties, often citing institutional continuity at specialized neuromuscular centers. The most common difficulties were inadequate physician knowledge of the disease (*n* = 7, 25.9%), insufficient clinical attention (*n* = 4, 14.8%), and access barriers including long appointment intervals (*n* = 4, 14.8%). Other difficulties included loss of multidisciplinary coordination (*n* = 3), provider discontinuity (*n* = 2), and the psychological challenge of adapting to an unfamiliar adult care environment (*n* = 3). One respondent identified hospital elevator access as their main difficulty. Only 5 of 29 transitioned patients (17.2%) reported receiving psychological support during the transition period.

#### Appointment adherence and access barriers

Appointment adherence after transition was unchanged in 69.0% of cases, decreased in 20.7%, and increased in 10.3%. The most common reasons for missed appointments were appointment scheduling system problems (*n* = 12, 41.4%) and transportation difficulties (*n* = 11, 37.9%), followed by difficulty finding an accompanying person (*n* = 3, 10.3%). Four respondents indicated no attendance problems.

#### Patient preferences and recommendations for improvement

Patient preferences and recommendations strongly favored structured, coordinated transition models. Twenty-five patients (86.2%) considered that a joint pre-transition clinic involving both pediatric and adult neurology teams would have been beneficial, and 28 (96.6%) endorsed the utility of a dedicated young adult clinic within the adult neurology department. Among the most valuable features of such a clinic, patients cited better communication, access to specialized disease knowledge, the availability of clinicians familiar with their specific condition, and age-appropriate emotional and psychological care.

When asked how the transition process could be improved, 5 of 29 patients and caregivers (17.2%) had no specific suggestion. The most frequent substantive recommendations were integration of multidisciplinary care under one structure with easier access to neuromuscular disease centers (*n* = 7, 24.1%), increased physician knowledge of and engagement with DMD in adult care (*n* = 5, 17.2%), joint pediatric and adult clinical meetings to support handover (*n* = 3, 10.3%), and psychological support during the transition period (*n* = 3, 10.3%). Other suggestions included continuity with the same treating physician where possible (*n* = 2), broader attention to quality of life beyond medical care (*n* = 2), and individualized engagement with patients and families (*n* = 2).

#### Patient goals and information needs

Among the 29 transitioned patients, the most commonly endorsed future goals were living independently (69.0%), going out more often (65.5%), spending more time with friends and family (51.7%), and making new friendships (48.3%). Continuing education and meeting new people were each endorsed by 34.5% and forming a romantic relationship by 27.6%. Among 30 free-text responses about what information patients and caregivers would have wanted during transition, the most commonly expressed needs were information about disease course and prognosis (*n* = 9), the transition process itself, including which specialties would be involved and how to prepare (*n* = 7), and treatment and medication management (*n* = 6). Other concerns emphasized the importance of communication between pediatric and adult teams (*n* = 3) and provider attitude and sensitivity (*n* = 2).

## Discussion

This is the first national-level characterization of DMD transition practice in Turkey, combining physician and patient perspectives across multiple regions. The Turkish health care system is a useful setting for this analysis because a national regulation sets the latest age for transfer to adult care, but no clinical framework specifies how transition should be conducted, so practice is shaped by individual centers and clinicians. A major finding from this study is that transition care in DMD across Turkey remains fragmented. Key gaps include limited readiness assessment, delayed discussions, poor patient and caregiver awareness of the transition process, and the absence of coordinated multidisciplinary structures. An effective transition is built on structured planning, coordinated handover, and post-transfer management [11, 20]. All three were unevenly delivered in our cohort. Planning, when present, was rarely formalized into individualized plans or readiness assessment. Handover was characterized by limited joint consultation between pediatric and adult teams and by patient preparation that most transitioned patients judged inadequate. Post-transfer management was largely untracked, with nearly half of physicians unable to estimate their loss-to-follow-up rate. Many of these gaps are not unique to Turkey. Late discussion, limited coordination between pediatric and adult teams, loss of multidisciplinary care after transfer, and a shortage of adult neurologists with neuromuscular expertise recur across other health systems [6, 13, 21, 22], which points to a structural rather than a local problem. However, there were some new observations in our study. Physicians and families gave different accounts of information transfer: every physician reported conveying some information, while many families believed nothing had reached the adult team, a gap in communication with families rather than a failure of documentation. Pediatric neurologists also described medico-legal exposure, rarely raised elsewhere, when they continue to manage adult-age complications without a formal handover.

A formal transition protocol with a designated lead person at each specialist center is the recognized first step in structuring transition care [20]. In our cohort, most transitions were managed by families rather than by clinicians or coordinators. Physicians strongly endorsed both a formal protocol and a dedicated coordinator role, the two recommendations that most directly address this gap. European rare disease centers with established programs typically assign coordinating tasks to nurses [23], and the regional DMD consensus places primary responsibility for the transition summary with the pediatric neurologist [19]. A division of labor between pediatric neurologists and a nurse or coordinator could organize these tasks without requiring new infrastructure and could be implemented with targeted training for existing staff.

Transition discussion in Turkey occurred generally later than international consensus recommends. For a smooth transition to happen, young people with DMD and their families should be made aware of transfer plans early, preferably before age 15 [13], with planning introduced between ages 12 and 14 [3], and patient knowledge-building from age 11 [19]. This gap between ideal and actual timing is not unique to Turkey: in the European NMD survey, 47.8% of respondents considered ages 15 to 16 ideal, but only 16.4% initiated at that age [6]. In our data, however, the pattern is sharper. Most physicians initiated transition at age 18, the majority of transitioned patients first heard about it at age 17 or later, and only a small minority of patients still in pediatric care had transition discussed with them at all. Late or absent discussion leaves little time for autonomy, self-management, and psychosocial preparation [6, 13]. Individualized written transition plans were also prepared by only a minority of physicians, close to the 23% reported in a US survey of young adults with DMD or BMD [17]. Routine introduction of transition planning before age 15, during regular clinic visits, is one of the most actionable changes in current practice, and one that can be implemented at the clinic level.

Coordination between pediatric and adult teams lagged behind information transfer. International consensus identifies combined pediatric and adult clinics, with all medical data accessible to both services, as a key element of transition [13]. In our cohort, all physicians reported transferring some form of information at the time of transition, most commonly through institutional electronic health records (80.6%) and written discharge summaries (61.3%). However, more than one third of patients and caregivers reported that no information had been conveyed to the adult team, reflecting limited communication with families during the handover rather than failure of the documentation transfer itself. Joint consultations between pediatric and adult neurology teams were held regularly by only a small minority of physicians, and most pediatric neurologists received no feedback from the adult team after transfer. By contrast, 61.2% of respondents in the European NMD survey reported regular joint meetings [6], pointing to what is possible in rare neuromuscular disease settings.

Multidisciplinary care often did not transfer with the patient. Roughly 44% of transitioned patients reported no specific gaps, usually because they remained at a specialized neuromuscular center. Among those who identified gaps, the most common was the loss of multidisciplinary coordination, particularly integrated cardiology and respiratory follow-up, both of which the European NMD survey identifies as critical concerns [6], given their central role in long-term DMD management. Consensus recommends a structured post-transition evaluation around six months after transfer [20]; that nearly half of our physicians could not estimate their loss-to-follow-up rate suggests this rarely happens.

The barriers the physicians identified in the study, including absence of a local transition program, parental preference for continued pediatric care, complexity of multisystem management, and insufficient training, have been similarly reported by others. The European NMD transition survey identified the need for multidisciplinary teams, lack of training, and insufficient financial support [6]; a 2024 US survey identified family pushback, difficulty finding adult providers, and communication gaps between teams [12]. Our findings refine this pattern: while adult neurologists in general were not perceived as scarce, the lack of adult neurologists with specific neuromuscular expertise emerged as a major concern, particularly outside major urban centers. The recurrence of similar barriers across three different health care systems suggests a structural rather than a local problem. Training emerged as a particularly persistent gap: in the European survey, lack of formal preparation was independent of clinician age and country of practice, and most respondents had built their transition knowledge through experience or informal mentorship [6]. Our findings fit this pattern, supporting structured training pathways as part of any future protocol.

Free-text responses surfaced additional concerns. Pediatric neurologists described medico-legal exposure when continuing to manage patients with adult-age complications in the absence of a formal handover pathway, an issue rarely raised in the international literature but consequential when no national framework exists. Patients identified appointment scheduling and transportation as the main reasons for missed follow-up, structural access barriers that disproportionately affect those with limited mobility. Psychological support was sparse, with only a small minority of transitioned patients receiving it during transition, and only a few specifically named it as a needed improvement. The future goals patients and caregivers reported, including social activities, independent living, friendships, continuing education, and romantic relationships, extended well beyond disease management and would be poorly served by any framework that addresses only the medical handover.

Effective transition has been described for years in the literature, yet our findings show it still begins only as transfer approaches. The elements of an effective transition are already set out in international consensus [3, 13, 19]; the task is to apply them earlier and in order. Because the fragmentation we found reflects the lack of a shared framework, the practical answer is a national transition protocol that each center runs through a named coordinator and trained staff and adapts to local conditions, placing transition with the clinical team rather than with families, who carried it themselves in our cohort. Such a protocol would replace individual judgment with an explicit timeline, drawing on what the international consensus recommends and adapting it to the Turkish setting: knowledge-building from around age 11, the need and timeline for transfer raised from age 14 [3, 19], and a readiness check between 14 and 16 using a validated tool such as the Got Transition Six Core Elements [3] or the Transition Readiness Assessment Questionnaire [24]. During the same period, the pediatric neurologist would identify a suitable adult center and prepare an individualized written plan with the team and family. Before transfer, a standard transition summary, or a clinical summary where full records cannot be shared, would replace the one-way handover seen in our cohort.

The two teams would meet in a combined pediatric and adult clinic, with at least two joint consultations from around age 16, as our respondents proposed, and a case conference reserved for the most complex patients [13]. After transfer, multidisciplinary follow-up would continue, preserving the cardiac and respiratory care most often lost in our cohort, with a review about six months later to confirm the patient has not dropped out [11, 13, 19]. In practice, transfer is best completed around age 18. The consensus allows longer, but pediatric care is oriented to patients under 18, and routine pediatric follow-up is often unavailable beyond that age, so the circular’s window up to 23 offers little real flexibility. Where local adult neuromuscular expertise is lacking, the circular permits continued pediatric follow-up, but a regional reference center or telehealth-supported local care usually offers a more sustainable solution. The exact timing of transfer would be agreed with the patient and family and guided by their clinical and psychosocial circumstances.

This study has several limitations. First, physicians who responded were actively involved in DMD care, so their answers reflect how transition is delivered in everyday practice rather than the impressions of clinicians who rarely see the disease. This also introduces a selection bias, and the responses may not represent DMD care in all neurology clinics throughout Turkey. Across both groups, our sample probably over-represents settings where general DMD care is better developed, so the gaps we report may understate those across the country. However, setting the physician responses alongside those of patients and families let us examine the same process from both sides and identify where the two perspectives agreed and where they differed. The DMD Families Association helped design and distribute the family survey, so the questions reflected the concerns that matter most to families. Second, although both surveys recruited volunteers, the participants in the two surveys were recruited in different ways. The physician survey was sent by cascade through two professional societies, while the family survey reached participants through the patient association and reference centers, so it likely drew families already linked to organized care, which may add an additional source of selection bias. Third, patient/family responses came from 24 cities but were unevenly spread, with some cities contributing more than others. Fourth, overall, the response rate to the patient/family survey was low, at 8.5%; however, because the invited population could not be restricted to eligible recipients, this is likely a lower bound rather than an exact rate. Finally, the patient/caregiver sample was small, with 29 patients in the transitioned subgroup, so these findings are descriptive rather than precise estimates and should not be generalized to all transitioned patients in Turkey. A sample of this size reflects the rarity of DMD. However, similar priorities emerged in the larger physician survey, which supports the descriptive reading despite the patient numbers.

In conclusion, transition care for DMD in Turkey remains fragmented and largely unstructured. Key gaps included limited readiness assessment, delayed transition discussions, poor patient and caregiver awareness of the handover, and the absence of coordinated multidisciplinary structures. Physicians and families identified the same priorities for improvement, providing a clear basis for a national transition framework. Such a framework should be adapted to the local health care context and account for the uneven distribution of adult neuromuscular expertise across the country. These are essential conditions for improving continuity of care for individuals with DMD in Turkey.

## Electronic supplementary material

Below is the link to the electronic supplementary material.

Supplementary Material 1

Supplementary Material 2

## Data Availability

The datasets generated and analyzed during the current study are not publicly available due to participant privacy considerations but are available from the corresponding author on reasonable request.
