# Supplementary material for: Transition from pediatric to adult neurology care in Duchenne muscular dystrophy: a national survey of patient and physician experiences
Source: Orphanet J Rare Dis. 2026 Jun 22;21:318. doi: 10.1186/s13023-026-04454-8 (PMC13576304; doi:10.1186/s13023-026-04454-8)
Supplement: Supplementary file 1 [file 13023_2026_4454_MOESM1_ESM.docx]

**Physician Survey: Transition from Pediatric to Adult Care in Duchenne Muscular Dystrophy**

**1. Please write your year of birth.**

**2. Please indicate your gender.**

☐ Female

☐ Male

**3. Please select your specialty.**

☐ Pediatric Neurology (Pediatrics background)

☐ Pediatric Neurology (Neurology background)

☐ Adult Neurology

☐ Other: _________

**4. Please write the city where you work.**

**5. Please write the name of your institution.**

**6. Which of the following best describes your institution?**

☐ Our center has been officially designated by the Ministry of Health as a *Neuromuscular Center.*

☐ Our center is not officially designated as a *Neuromuscular Center,* but it has an *active Neuromuscular Outpatient Clinic.*

☐ Neuromuscular patients are seen routinely within pediatric neurology/neurology practice.

☐ Other: _________

**7. What type of institution do you work in?**

☐ Children’s Hospital

☐ Adult Hospital

☐ Both (provides pediatric and adult services)

☐ Other: _________

**8. How much time do you usually allocate for an outpatient visit a patient with DMD?**

☐ Less than 15 minutes

☐ 15–30 minutes

☐ More than 30 minutes

☐ Other: _________

**Section 2: Transition Policy and Awareness**

**9.** Are you aware of the Turkish Ministry of Health circular requiring that patients with childhood-onset diseases who need continued care into adulthood be transferred to an adult specialist by age 23?

☐ Yes

☐ No

**10.** Does your institution have a structured transition program or guideline for neurological diseases that start in childhood and continue into adulthood?

☐ Yes

☐ No

☐ I’m not sure

**11.** Is there a specific transition procedure for individuals of DMD in your institution? How is it currently managed?

☐ No formal procedure

☐ Periodic meetings between pediatric and adult neurologists

☐ Joint clinics held periodically by pediatric and adult neurologists

☐ Patients/parents are informed about communication channels and procedures for visiting adult care centers

☐ Personal communication between pediatric and adult neurologists

☐ I’m not sure

☐ Other: _________

**12.** At what age does your institution typically begin the transition process?

**13.** In your opinion, at what age should the transition ideally begin?

**14.** At what age are patients typically fully transferred to adult neurology services?

**Section 3: Follow-Up and Monitoring**

**15.** Is there a systematic record (list, software, etc.) for tracking patients in transition?

☐ Yes

☐ No

☐ I’m not sure

**Section 4: Transition Readiness Assessment**

**16.** Do you use any tool/form/interview to assess readiness for transition?

If yes, please specify which tool.

☐ No

☐ Other: _________

**17.** Are appointments conducted (entirely or partially) without parents in the exam room during the transition preparation phase?

☐ Yes

☐ No

☐ Other: _________

**Section 5: Transition Planning**

**18.** Is an individualized transition plan (medical summary, social planning, etc.) prepared for each patient?

☐ Yes

☐ No

**19.** How is the patient’s medical information transferred to the adult physician during the transition? *(Multiple selections allowed)*

☑ Institutional electronic medical record system

☑ Physical documents/discharge summaries

☑ Telephone communication

☑ No transfer is made

☑ Other: _________

**20.** Are the views of the patient and family included in the transition planning process?

☐ Yes

☐ No

**Section 6: Transfer to Adult Care – Completion and Feedback**

**21.** Are joint case discussions or ward rounds conducted between pediatric and adult neurology teams before transition?

☐ Yes, regularly

☐ Sometimes

☐ No

**22.** Do you or your team continue to follow the patient temporarily after transfer? *(If you are an adult neurologist, please leave blank.)*

☐ Yes, for a limited period

☐ No

**23.** Do you receive feedback from the adult team regarding patient follow-up after transfer? *(If you are an adult neurologist, please leave blank.)*

☐ Yes

☐ No

**24.** In your opinion, how high is the rate of patient loss to follow-up after transition?

☐ High

☐ Low

☐ I don’t know

**Section 7: Clinical Barriers (Likert Scale)**

**25.** Please indicate the extent to which you agree that the following factors create barriers in the transition process for your patients:

*(1 = Strongly Disagree, 5 = Strongly Agree)*

1. Lack of multidisciplinary team/communication in the adult system
2. Lack of communication between adult and pediatric teams
3. Limited number of adult neurologists
4. Insufficient knowledge among adult neurologists about childhood-onset diseases and their adult care
5. Lack of guidelines for adult care of childhood-onset diseases
6. Limited willingness among adult neurologists
7. Difficulty obtaining timely appointments in adult neurology clinics
8. Difficulty accessing pediatric medical records
9. Lack of adapted clinical settings for young adults with special needs (autism, intellectual disability, behavioral issues, wheelchair users, etc.)
10. Complexity of multisystem pediatric-onset diseases
11. Parents/caregivers preferring to remain in pediatric care
12. Absence of a locally established transition program
13. Lack of financial support for transition programs (e.g., coordinator positions)
14. Lack of awareness regarding the need and implementation of transition programs
15. Insufficient training/knowledge about transition

**Section 8: Recommendations (Likert Scale)**

**26.** Please indicate how important you consider the following recommendations for strengthening the transition process:

*(1 = Not important, 5 = Very important)*

1. Joint consultations/meetings between pediatric and adult neurology teams
2. Use of national recommendations for transition from pediatric to adult care
3. Appointment of a dedicated transition coordinator
4. Active involvement of patients and families in transition planning
5. Inclusion of patient and caregiver perspectives and preferences in transition planning
6. Having a formal transition protocol

**Other (please specify):** _________

**Open-Ended Questions**

**27.** How satisfied are you with the transition process in your institution?

☐ Completely satisfied

☐ Partly satisfied

☐ Not satisfied at all

**28.** Please describe the transition process in your institution.

**29.** What are your suggestions for improving the transition process in neuromuscular disorders?

**Patient and Caregiver Survey: Transition from Pediatric to Adult Care in Duchenne Muscular Dystrophy**

**Section 1 – Respondent Information**

Who is completing this survey?

☐ I am completing it myself. (I am a person diagnosed with DMD or BMD)

☐ I am completing it on behalf of the patient. (Parent or primary caregiver)

**Section 2 – Participant Details (Anonymous Identification)**

1. First two letters of the patient’s first name:

(e.g., Ahmet → **Ah**)

2. First two letters of the patient’s last name:

(e.g., Yılmaz → **Yı**)

3. Patient’s year of birth:
4. City of residence:

**Section 3 – Diagnosis and Education**

5. What is the diagnosis?

☐ Duchenne Muscular Dystrophy (DMD)

☐ Becker Muscular Dystrophy (BMD)

☐ Other: _________

6. What is the patient’s current education or occupational status? *(Tick all that apply)*

☑ Currently in secondary school

☑ Currently in university

☑ Receiving support from a special education and rehabilitation center

☑ Working full time

☑ Working part time

☑ Not working

☑ Working online/remotely

7. What is the highest level of education the patient has completed?

☐ Primary school

☐ Middle school

☐ High school

☐ Vocational school / Associate degree

☐ University / Bachelor’s degree

☐ Master’s degree

☐ Doctorate or higher

8. Is there anyone else in your family with the same diagnosis?

☐ Yes

☐ No

9. Does the patient have a family physician or general practitioner who regularly follows up?

☐ Yes → Move to Question 10

☐ No → Move to Question 11

10. Please indicate which statements apply to your family physician: *(Tick all that apply)*

☑ My family physician is well informed about my condition

☑ My family physician knows and prescribes my medications

☑ Provides advice on healthy lifestyle habits

☑ Provides advice on vaccinations

☑ Provides advice on mental or psychological concerns

☑ Maintains regular communication with my neurologist/pediatric neurologist

**Section 4 – Knowledge and Consultations**

11. Does the patient know the names and doses of the medications used for this condition?

☐ Yes

☐ No

12. Has the pediatric neurologist ever met with the patient **alone** (without a family member present) to discuss DMD or BMD during clinical visits?

☐ Yes → Move to Question 13

☐ No → Move to Question 14

13. If yes, at what age did the first one-on-one consultation take place?

**Section 5 – Current Medical Follow-Up**

14. Which type of clinic or specialist currently provides the patient’s follow-up care?

☐ Pediatric clinic / Pediatric neurologist → Move to Question 15

☐ Adult clinic / Adult neurologist → Move to Question 16

15. Has your pediatric neurologist or care team ever discussed the **transition to adult neurology** with you?

☐ Yes

☐ No

☐ Not sure

16. Before transferring to adult care, had the patient ever visited the hospital or clinic where the adult neurology team works?

☐ Yes

☐ No

17. Before transition, did the patient ever meet the adult neurology team alone?

☐ Yes

☐ No

18. How old was the patient when the topic of transition to adult care was first discussed?

☐ 12–14 years

☐ 15–16 years

☐ 17 years or older

☐ Not known

**Section 6 – Transition Preparation**

For each topic below, please indicate whether it was discussed with the patient before transition:

| **Topic** | **Yes** | **No** |
| --- | --- | --- |
| Which hospital the patient would transition to | ☐ | ☐ |
| Which healthcare teams would be involved in future follow-up | ☐ | ☐ |
| Genetic counseling | ☐ | ☐ |

19. Do you think your preparation for transition during pediatric care was sufficient?

☐ Yes

☐ No

20. How was the transition carried out?

☐ Structured/planned process (information about institutional transition policy, periodic readiness assessment, detailed transfer documents prepared, etc.)

☐ Discussion with pediatric neurologist

☐ Pediatric neurologist sent a referral letter/discharge summary to the adult neurologist

☐ Pediatric neurologist gave a referral letter/discharge summary directly to us

☐ No information was transferred

☐ Other: _________

21. Who do you think coordinated the transition process?

☐ Our family (ourselves)

☐ Pediatric neurologist

☐ Family physician

☐ Nurse

☐ Other: _________

22. Did the patient receive any **psychological support** related to the transition process?

☐ Yes

☐ No

**Section 7 – Concerns and Expectations**

23. Did the patient have any concerns about transitioning to adult neurology follow-up?

☐ Yes → Move to Question 24

☐ No → Move to Question 25

24. Please describe your concerns about moving to adult care. *(Open-ended)*
25. What kind of information or support would you have liked to receive during this process? *(Open-ended)*

**Section 8 – Self-Care and Medications**

26. Does the patient schedule neurology appointments independently?

☐ Yes

☐ No

If yes, at what age did this begin?

27. Does the patient meet with the neurologist alone (for part or all of the visit)?

☐ Yes

☐ No

If yes, from what age?

28. Who is responsible for ensuring that medications are taken regularly?

☐ The patient

☐ Family/caregiver

**Section 9 – Sources of Information and Support**

29. Where do you obtain information about your condition and treatment? *(Tick all that apply)*

☑ Family members or relatives

☑ Pediatric neurologist

☑ Adult neurologist

☑ Family physician

☑ Internet websites

☑ Patient or family associations

☑ Other: _________

30. Before transferring to adult neurology, would it have been helpful to meet a member of the adult neurology team during a joint pediatric–adult clinic?

☐ Yes

☐ No

31. Please explain why you think so. *(Open-ended)*

**Section 10 – Social Goals**

32. Do you think having a dedicated **Young Adult Neurology Clinic** within the adult department would be beneficial?

☐ Yes

☐ No

33. Please explain why. *(Open-ended)*
34. Were there any specific services or supports available in pediatric care that you felt were **lacking in adult neurology services**?

(e.g., collaboration with other professionals such as psychology, social work, nutrition, cardiology, respiratory therapy, physiotherapy, etc.)

35. How did this affect you? *(Open-ended)*
36. What were the **main challenges** you faced when transitioning from pediatric to adult neurology services? *(Open-ended)*
37. How can we make the transition process better for young adult patients in the future? *(Open-ended)*

**Section 11 – Comparison of Pediatric and Adult Follow-Up**

38. Comparing your experiences, which follow-up period do you view more positively?

☐ Pediatric follow-up

☐ Adult follow-up

☐ Both are similar

39. If there is a difference, what do you think causes it?

(e.g., physician communication, coordination, information sharing, appointment scheduling, etc.)

40. Comparing your adherence to appointments and follow-ups between childhood and adulthood, how would you describe it?

☐ My adherence decreased

☐ My adherence did not change

☐ My adherence improved

41. If you have missed appointments, please indicate the reasons: *(Tick all that apply)*

☑ Transportation difficulties

☑ Problems in appointment system

☑ No available companion

☑ Work/school/time conflict

☑ Forgetting to make an appointment

☑ Not feeling the need for follow-up

☑ Other: _________
