# Supplementary material for: Transition from pediatric to adult neurology care in Duchenne muscular dystrophy: a national survey of patient and physician experiences
Source: Orphanet J Rare Dis. 2026 Jun 22;21:318. doi: 10.1186/s13023-026-04454-8 (PMC13576304; doi:10.1186/s13023-026-04454-8)
Supplement: Supplementary file 2 [file 13023_2026_4454_MOESM2_ESM.docx]

**TRANSITION FROM PEDIATRIC TO ADULT NEUROLOGY CARE IN DUCHENNE MUSCULAR DYSTROPHY: A NATIONAL SURVEY OF PATIENT AND PHYSICIAN EXPERIENCES**

**Supplementary Table 1. Patient-reported post-transition outcomes, autonomy, and preferences (n=29 transitioned patients)**

| **Variable** | **n** | **%** |
| --- | --- | --- |
| ***Educational and vocational status*** | | |
| Not working or not in education | 11 | 37.9 |
| Attending a special education or rehabilitation center | 8 | 27.6 |
| In university education | 4 | 13.8 |
| In secondary education | 3 | 10.3 |
| In university education with special education support | 2 | 6.9 |
| Working full-time | 1 | 3.4 |
| ***Highest educational attainment*** | | |
| High school | 14 | 48.3 |
| Middle school | 7 | 24.1 |
| Primary school | 4 | 13.8 |
| Associate degree | 2 | 6.9 |
| University (Bachelor's) | 2 | 6.9 |
| ***Patient autonomy in adult care*** | | |
| Manages own medications | 12 | 41.4 |
| Books own appointments | 8 | 27.6 |
| Consults neurologist alone | 3 | 10.3 |
| ***Sources of disease and treatment information ᵃ*** | | |
| Patient groups or family associations | 22 | 75.9 |
| Internet | 17 | 58.6 |
| Child neurologist | 16 | 55.2 |
| Adult neurologist | 9 | 31.0 |
| Family or relatives | 2 | 6.9 |
| ***Preferences for future transition support*** | | |
| Joint pre-transition clinic would have been beneficial | 25 | 86.2 |
| Young adult clinic in adult neurology would be useful | 28 | 96.6 |

ᵃ Multi-select item; percentages may exceed 100%.
